# The Impact of Recess on Chronic Stress Levels in Elementary School Children †

**DOI:** 10.3390/children12070865

**Published:** 2025-06-30

**Authors:** Deborah J. Rhea, Kelsey Kirby, Dennis Cheek, Yan Zhang, G. Kate Webb

**Affiliations:** 1 Kinesiology Department, Texas Christian University, Fort Worth, TX 76129, USAg.kate.webb@tcu.edu (G.K.W.); 2 Nursing Department, Texas Christian University, Fort Worth, TX 76129, USA; d.cheek@tcu.edu; 3 Applied Health Sciences Department, Texas Christian University, Fort Worth, TX 76129, USA; yan.zhang@tcu.edu

**Keywords:** chronic stress, hair cortisol concentration, children, social skills, physical activity, recess, outdoors, play, mental health

## Abstract

Background: Over the last 30 years, stress and anxiety in children have risen exponentially, especially as a result of school expectations. As no studies have examined the effect of increased outdoor recess on children’s chronic stress levels, this preliminary work focused on comparing hair cortisol concentration (HCC) scores of elementary children who received varied amounts of daily recess. Methods: HCC scores were collected from 4th grade elementary children (N = 130) from three intervention schools (45 min daily recess) (N = 64; M = 35; F = 29) and three control schools (30 min daily recess) (N = 66; M = 31; F = 35). Results: A two-way ANOVA, F(1, 123) = 5.47, *p* = 0.021, ω^2^ = 0.034, showed that the intervention group reflected lower HCC levels (marginal M = 5.69, 95% CI [−1.92, 13.30]) than the control group (marginal M = 18.22, 95% CI [10.83, 25.61]). Post hoc analysis revealed an estimated marginal mean difference of −12.53 (SE = 5.36, *p* = 0.021). Sample *t*-tests compared HCC levels against the pre-COVID normative value of 7.5 pg/mg. The intervention group HCC mean (M = 5.85, SD = 6.26) was significantly lower than the normative value, t(61) = −2.08, *p* = 0.042. The control group’s mean HCC (M = 18.22, SD = 41.39) was significantly higher than the normative value, t(64) = 2.09, *p* = 0.041. Conclusions: Increasing daily recess may contribute to lower chronic stress levels among 4th grade children. With obesity and mental health disorders on the rise and American students’ academic performance on the decline, these pilot results cannot be overlooked or dismissed.

## 1. Introduction

Decades ago, the U.S. practiced a simple philosophy based on developmentally appropriate practices for children [1,2]. From home life to the school setting, children were expected and allowed to play daily and often [3]. Adults recognized that play, especially outdoor play, was essential for healthy and happy children. In the school setting, children experienced 60 min of daily outdoor recess and at least 45 min of daily physical education [3,4], in addition to the outdoor play experiences they had at home [4]. As a result, families raised happy, healthy, and resilient children who became well-adjusted, healthy, and resilient adults [3].

This is not the case today. Over the last 30 years, adults have created a school environment that is counterintuitive to a child’s ability to learn. The school setting has shown a sharp decline in recess and physical education levels in order to focus almost entirely on increased classroom time and standardized test scores [4]. The absence of outdoor play puts children at a high risk of falling behind age-appropriate milestones, including brain development and function [5], simple and complex movement patterns [6,7], social skills with peers, and mental/emotional stability [3,8,9]. The effects of the COVID-19 pandemic compounded these developmental deficits. Children began to show mental health declines, with increased anxiety and depression levels, after the pandemic when compared to pre-COVID populations [10,11]. The combination of decreased play in childhood and a world pandemic that separated children from their daily school experiences has produced more need for increased daily outdoor play.

The results of limited outdoor play have been realized. Over the past 20 years, the CDC has seen a rise in children who are healthy at birth, but then develop an increasing number of non-communicable disease (NCD) health risks as they mature into adolescents and adults [12]. The factors contributing the most to NCDs are that 81% of adolescents lack physical activity and one in five children over the age of five are obese with this number rising yearly [12]. These contributions have led to one in six children between the ages of two and eight being diagnosed with mental, behavioral, or developmental disorders related to chronic stress [12]. Schools have further exacerbated the chronic stress problem [13]. Almost 70% of elementary school children self-report feelings of stress and anxiety from school requirements, such as high expectations on standardized tests [12]. This increased stress in children leads to increased recurring headaches and stress-related symptoms, such as physical fatigue, the inability to relax, anxiety, fear, and restlessness [14]. Furthermore, children diagnosed with health problems at an early age may have much higher chronic stress risk throughout childhood and adolescence [15,16]. As children mature into adulthood, the negative health outcomes caused by chronic stress progress into high blood pressure, high cholesterol, obesity, and other chronic diseases [17,18,19,20,21].

As a result of these trends, a growing number of studies have focused on identifying the most accurate way to assess stress levels in children [22]. Of the existing physiological stress indicators, cortisol is considered the most reliable biomarker for measuring physiological stress levels [22]. To date, most studies have typically measured cortisol in saliva, urine, and serum samples [22,23]. In children, researchers have most commonly measured cortisol through saliva due to the nonpainful sampling and ease of storage [24]. However, these methods reflect acute or short-term cortical hormone levels, which often have daily fluctuations.

In contrast, hair cortisol concentration (HCC), a promising long-term analysis technique, is accessible to investigators and provides a reliable and valid three-month average index of cortisol secretion [25,26]. This makes HCC analysis a very attractive measure for determining chronic stress [22,27], especially in children, for which rapid, noninvasive sampling procedures are essential. Researchers have widely used HCC to approximate chronic physiological stress responses in adults [28], but the measure is only recently emerging in children and has shown varying results [24]. Despite the lack of HCC reference norms and different HCC analysis techniques for different age groups, HCC information within and across children seem to be highly comparable [24]. Of the six reviewed studies examining elementary-aged children, high HCC variability was found among age and sex based on the HCC extraction method used [24]. Higher HCC levels have been found using an enzyme-linked immunoassay (ELISA) versus liquid chromatography-mass spectrometry (LCMS) [27]. Using ELISA, scores have ranged from 0.07 to 369.60 pg/mg depending on age and geographic location [29,30,31,32]. In general, global findings have shown the lowest hair cortisol levels in six-year-old children, after which HCC levels increase gradually until the age of 18 years [33]. U.S. studies have recorded higher HCC pg/mg levels than other countries across age levels [33]. Finally, in one of the largest studies published, Noppe et al. 2014 [23] provided specific cortisol averages by age level: 6–7 year olds’ average cortisol was 5.8, 8–9 year olds’ average cortisol was 6.8 pg/mg, and the 9–10 year olds’ average cortisol was 8.5 pg/mg [23]. These pre-COVID studies focused on average and healthy HCC stress levels of children [24,29,30,31,32], but post-COVID detriments of stress levels in children are yet to be studied.

The LiiNK Project^®^ (Let’s inspire innovation ‘N Kids^®^), a longitudinal recess intervention, has successfully implemented four 15 min unstructured, outdoor play breaks, known as recess, in elementary schools daily over a six-year period, in order to produce a healthier environment for children to navigate [4,6,7,8,19,20]. Typically, all teachers per grade level take their students outside simultaneously four times daily for 15 min each time, and they cannot withhold recess for tutoring or punishment. In some instances, as children enter the 4th and 5th grades, the school may alter the 60 min of recess to 45 min due to the pressure of standardized test outcomes.

No studies have shown the influence of increased recess (free play) in elementary school environments on a child’s chronic stress levels. Therefore, this preliminary chronic stress work was focused on comparing HCC scores between 4th grade children who received the LiiNK intervention (45 min of recess) and the same age group in a typical elementary school environment from the same school district (30 min or less of recess). The first hypothesis states that the 4th grade children in the intervention group would show significantly lower levels of chronic stress than the typical school children, while not differing within recess groups by sex. The second hypothesis states that children in the intervention group would be similar in terms of cortisol averages to the pre-COVID normative standards, while the typical school children would be higher in terms of their cortisol averages than the pre-COVID normative standards [23].

## 2. Materials and Methods

### 2.1. Participants

Elementary school children (N = 130) volunteered from 4th grade classrooms (9 yrs = 98, 10 yrs = 29) within six North Texas public elementary schools, all within the same school district. The six schools included three intervention schools (N = 64; M = 35; F = 29) and three control schools (N = 66; M = 31; F = 35). The intervention school children received 45 min of recess and a 15 min character development lesson daily. The control school children received 30 min of recess and no character development lessons.

### 2.2. Measures

#### 2.2.1. Demographic Information

Age (date of birth), sex, and teacher name were shared with the LiiNK primary investigator (PI) through each school’s administrative assistant. This information helped the LiiNK team schedule/locate the children with parental consent on the data collection day.

#### 2.2.2. Chronic Stress Level

Hair cortisol concentration (HCC) measures chronic stress levels in children and adults and has repeatedly been shown to be an accurate and reliable estimate of long-term cortisol secretion [28,34]. Historically, three months is considered a reliable representation of chronic stress levels. Each centimeter of hair analyzed determines the average stress response, or cortisol secretion, over a month’s period. Therefore, each sample was at least 3 cm in length. A hair sample, containing 30–50 strands, which is smaller than half the width of a pencil, was collected from the back of the head underneath the top layer, as it was protected from sun exposure. The laboratory analysis used in this study was an enzyme-linked immunosorbent assay (ELISA) to evaluate the hair samples for cortisol concentration. Since each individual’s hair has a different density, the weights of hair samples during laboratory analysis varied. Genetic-type testing is not possible since the hair follicle is not collected. 

### 2.3. Procedures

Ethics approval was granted by the university’s institutional review board (IRB) for this study. School personnel, including administrators and teachers, approved the study prior to parental notification of the hair cortisol study.

Once parents/legal guardians received the consent letter through email detailing the hair collection criteria, including a video link of the process to assess chronic levels of cortisol, the next step was for the teacher to collect all returned consent forms. Once collected, the school would contact the primary investigator (PI) to pick up all forms. Once the PI had collected all consent forms from each school, the next step was to enter all information into a Microsoft Excel file, and save it in a protected university-assigned drive. All hard copies of the consent forms were filed in a locked file cabinet for future reference.

Inclusion criteria for this study were (1) parental consent, (2) hair that was at least 1 inch in length, and (3) child assent given for collecting a hair sample. Diseases that could influence the glucocorticoid levels (especially Addison’s disease and Chushing’s disease), including anxiety and attention-deficit/hyperactivity disorder, and pharmacological treatments (administered by any method, i.e., oral, inhaled, or topical) that could influence cortisol levels, including antipsychotics, ADHD medications, and corticosteroids, were not excluded from data collection. Additionally, any initially acceptable child who did not assent to the collection of hair, did not have sufficient hair length, or was absent on the day of data collection was excluded from the study. 

Prior to data collection, the PI trained five other team members, also IRB-approved, on how to operate equipment and properly complete procedures. Due to rotating schedules, team members were provided written procedures on the collection and storage process for the hair samples as well.

#### 2.3.1. Hair Cortisol Concentration (HCC) Collection

Children with parental consent and that met the inclusion criteria were asked to enter the designated school gym collection area (coaches’ office) one at a time while the rest of the children in the physical education class were participating in teacher-led activities. The PI gave the hair collection protocol instructions following a verbal explanation of the procedure. This explanation included a visual demonstration of the amount of hair that would be taken and how it was collected to assess a stress hormone measurement called cortisol. Following verbal explanation, each child was provided the opportunity to ask questions, as well as confirm or deny their assent. Any child who denied their assent or voiced feeling uncomfortable was given permission to return to class. 

If the child assented, the child signed and dated the form confirming the assent. The PI prepared an 8 × 10 in sheet of aluminum foil, a labeled Manilla envelope, painter’s tape, and a permanent marker nearby. Then, the child’s hair was measured with a ruler from scalp to end, by pulling the hair gently. With clean, salon-grade scissors, a comb, and clips, a non-latex-gloved researcher combed and parted the hair horizontally along the posterior vertex of the scalp between the tips of the ears and made a clean, straight cut as close to the scalp as safely possible.

After the hair was cut, the end closest to the scalp was attached to the prepared sheet of aluminum foil by using the painter’s tape. The tape attached the hair to the foil below the 3–4 cm point on the hair, and a permanent black marker was used to indicate the scalp end of the hair with an arrow drawn on the foil. Then, the foil was neatly folded on all sides to ensure the hair could not escape. It was then placed inside the Manilla envelope, sealed, and labeled with the child’s ID number, the collection date, and the initials of the PI. The packaged hair samples were stored at room temperature inside an airtight storage container, within the PI’s university locked office, until the cortisol extraction was completed.

#### 2.3.2. Hair Cortisol Extraction

Once all hair samples were collected, they were transported to the lab where the samples were divided into groups of 36, as each microtiter plate tests 36 samples, in addition to the NSB (control) wells. After the samples were divided into the four plate groups, the reagents and sample placements were prepared for the first plate. From there, the first 36 hair samples were individually measured and trimmed down to exactly 3 cm in length, from the scalp end. Each sample was then placed in a disposable, polypropylene tube, weighed, washed, and dried. After the samples had 48 h to dry, two steel beads were added to each sample, pulverized, and then left to incubate in methanol for 24 h. Next, the samples were centrifuged at 10,000 rpm into a pellet, which evaporates the methanol from the tube. Finally, cortisol was extracted from the sample and placed into the microtiter plate for analysis using a high-sensitivity enzyme immunoassay (ELISA) kit. After the raw data from the ELISA were determined, control ranges were computed using optical density, percent bound, and control concentrations to form a 4-parameter non-linear regression curve fit. This standard curve is different for each plate since each ELISA kit comes with its own vials of solutions to be used with the designated plate.

### 2.4. Data Analysis

Descriptive statistics (e.g., frequencies, means, and standard deviations) were calculated to address Hypothesis 1 by summarizing demographic variables (age and sex) and hair cortisol concentration (HCC) levels for the intervention and control groups. Once means and standard deviations were calculated, if a child’s cortisol level was more than two deviations away from the mean, that child was excluded from any further results. To test the first hypothesis, a two-way analysis of variance (ANOVA) was conducted, with group (intervention vs. control) and sex (male vs. female) as independent factors and HCC levels as the dependent variable. This analysis examined the main effects of group and sex, as well as the group × sex interaction, to explore whether the relationship between group and HCC levels differed by sex.

Tests for normality and homogeneity of variance were performed to evaluate the assumptions of the ANOVA model. While violations of these assumptions were observed, the analysis proceeded with caution, and results were interpreted accordingly. Post hoc analyses were conducted to further explore significant main effects and interactions.

To address Hypothesis 2, one-sample *t*-tests were conducted to compare the mean HCC levels of the intervention and control groups against pre-COVID normative cortisol standards (7.5 pg/mg). We used 7.5 pg/mg as the comparison value for normative hair cortisol concentration (HCC) because it represents the midpoint between the reported averages for 8–9 years (6.8 pg/mg) and 10–14 years (8.5 pg/mg) based on Noppe et al. (2014) [23]. Since the study population includes 9–10-year-old children, this midpoint serves as a reasonable approximation of the expected normative HCC level for this specific age group. The alpha level was set as 0.05 for all statistical tests.

## 3. Results

### 3.1. Descriptive Statistics

A total of 195 children participated in this study. Sixty-five of those children were excluded due to dissent, hair limitations for sampling, or absence on the day of data collection. Therefore, this study yielded a sample size of 130 children (intervention = 64, control = 66). The intervention children had a slightly lower average age (M = 9.17) than the control school children (M = 9.34). Eighty-four percent of the intervention children were 9 years old and 16% were 10 years old, whereas 70% of the control children were 9 years old and the other 30% were 10 years old. The two groups were not significantly different by age for this 4th grade sample (*p* > 0.05); therefore, age was not included as a factor in the comparison of the hair cortisol level.

**Figure 1:**
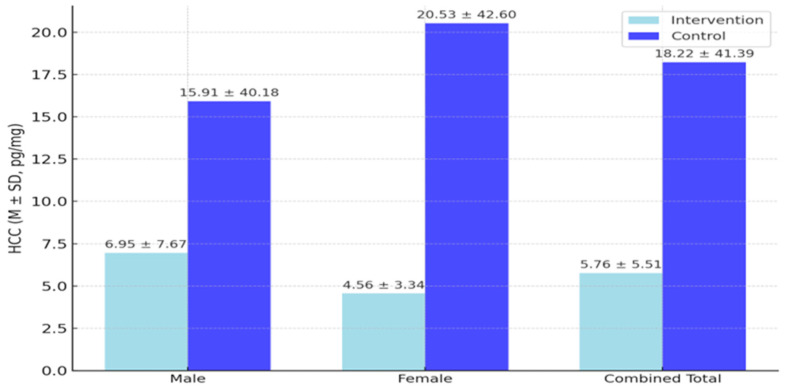
HCC levels by group and sex.

The lowest cortisol concentration found in any hair sample was 0.24 picogram per milligram (pg/mg), whereas the highest cortisol concentration was 221.04 pg/mg (M = 12.27, SD = 30.38). The control group children’s average cortisol concentration was found to be higher than the overall sample average (M = 18.220, SD = 41.39). Meanwhile, the intervention group children had an average cortisol concentration well below the overall sample average (M = 5.76, SD = 5.51). Figure 1 presents the descriptive statistics for HCC scores by group and sex, along with combined totals for the intervention and control groups.

### 3.2. Hair Cortisol Differences

A two-way ANOVA examined the effects of group (LiiNK intervention vs. control) and sex on hair cortisol concentration (HCC). A significant main effect of group was observed, F(1, 123) = 5.47, *p* = 0.021, ω^2^ = 0.034, with lower HCC levels in the intervention group (marginal mean M = 5.69, 95% CI [−1.92, 13.30]) compared to the control group (marginal mean M = 18.22, 95% CI [10.83, 25.61]). Marginal means are model-based estimates adjusted for the effects of both group and sex, providing a balanced comparison. Post hoc analysis revealed an estimated marginal mean difference of −12.53 (SE = 5.36, *p* = 0.021), Cohen’s d = 0.41, indicating a small to moderate between-group effect based on Cohen’s benchmark [35].

**Figure 2:**
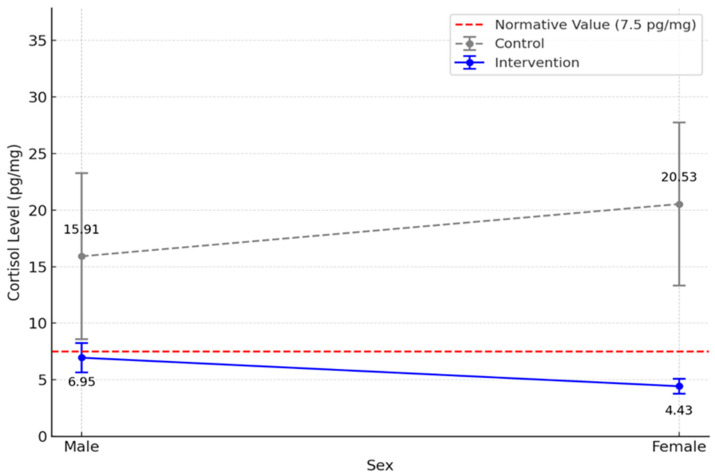
Post hoc cortisol level comparisons by sex and group.

The main effect of sex was not significant, F(1, 123) = 0.038, *p* = 0.845, and no significant interaction between group and sex was found, F(1, 123) = 0.507, *p* = 0.443. However, the marginal means suggest a trend: control group females had higher HCC levels (M = 20.53, SD = 42.60) than males (M = 15.91, SD = 40.18), Cohen’s d = 0.11), whereas in the intervention group males had higher HCC levels (M = 6.95, SD = 7.67) than females (M = 4.43, SD = 3.35), Cohen’s d = 0.41). Both sex effects are small to moderate; neither reached statistical significance. This trend is illustrated in Figure 2, despite no significant interaction. Therefore, the data did not lead to the rejection of Hypothesis 1.

Levene’s test indicated a violation of the homogeneity of variance, F(3, 123) = 6.62, *p* < 0.001, warranting caution in interpreting results.

To test the second hypothesis, one-sample *t*-tests were conducted to compare the hair cortisol concentration (HCC) levels of the intervention and control groups against the pre-COVID normative value of 7.5 pg/mg.

**Figure 3:**
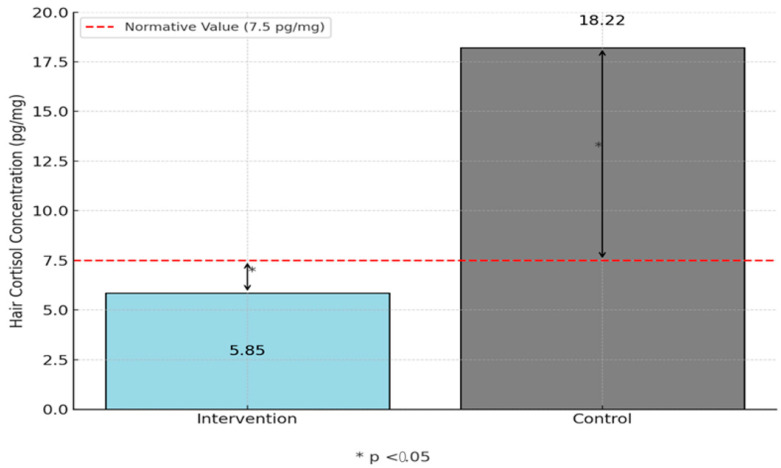
HCC level comparisons between groups against the normative value.

For the intervention group, Figure 3 shows the mean HCC (M = 5.85, SD = 6.26) was significantly lower than the normative value, t(61) = −2.08, *p* = 0.042, Cohen’s d = –0.26, indicating reduced chronic stress with a small effect. In contrast, Figure 3 also shows the control group’s mean HCC (M = 18.22, SD = 41.39) was significantly higher than the normative value, t(64) = 2.09, *p* = 0.041, reflecting elevated chronic stress in children without the intervention, but with a small effect. Therefore, the data did not lead to the rejection of Hypothesis 2.

## 4. Discussion

To date, very little research has assessed chronic stress using hair cortisol concentration (HCC) in children of any age. The purpose of this study was to measure the effect of a recess intervention on children’s chronic stress levels.

### 4.1. Group Cortisol Concentrations

The intervention group 4th grade HCC levels were found to be significantly lower than the control group 4th grade levels, which is where the results of this study reflect a preliminary intervention positive impact. The implementation of 45 min of daily recess, defined as unstructured outdoor play, showed that children need at least three 15 min breaks a day to de-stress. Thirty minutes daily does not seem to be enough. Previous research has shown the children with more recess opportunities throughout a school day achieved significantly higher levels of physical activity [19,20], while also improving physical, psychological, and cognitive health [5,6,7,8,9], all of which aid in lowering chronic stress levels [36]. It is not surprising, then, that children who have increased recess also have decreased stress levels as physical activity opportunities increase through recess [19,20]. Conversely, increased seat time in school without recess throughout the day may increase stress responses brought about by environmental stressors, such as the aftermath of the COVID-19 pandemic [10,11,17].

Recess has social implications for decreasing stress levels in childhood as well. These include ample opportunities to participate in and strengthen communication with peers or adults regarding thoughts and feelings. Studies show that without this frequent opportunity to connect socially, even healthy children with no pre-existing psychiatric diagnosis report increases in anxiety and decreases in attention, which are indications of unhealthy stress levels associated with isolation [37]. As childhood and adolescence are pivotal periods for establishing emotional health, whole-child interventions such as those focused on recess offer promising opportunities for increased physical and social–emotional health, as well as emotional regulation [8,38].

### 4.2. Group by Sex

While the group × sex interaction was not statistically significant, the descriptive data and interaction plot reveal a notable trend in cortisol levels between boys and girls in the intervention and control groups. In the control group, girls exhibited higher cortisol levels compared to boys, suggesting greater chronic stress among girls. In contrast, in the intervention group, boys had higher cortisol levels than girls, indicating differing responses to the intervention.

This pattern highlights potential sex-based differences in how children experience and regulate chronic stress. Research trends show males need physical activity and recess more than girls because of their need for movement [39]. Conversely, research shows that girls begin to decrease in physical activity levels sooner than boys [40]. In this study, girls seem to be more heavily influenced by having at least 15 additional minutes of recess daily than the boys in both groups. This reduction in chronic stress for the intervention girls over the control girls may be due to females, in general, participating in less physical activity from a young age; therefore, they are more responsive to the 15 min increased recess dosage. Additionally, girls may be more susceptible to stress due to differences in perception, coping mechanisms, or environmental influences [39]. The intervention also reduced cortisol levels for the boys, but overlapping data points with boys in the control group might explain the lack of statistical significance for the interaction effect. These findings support the LiiNK intervention’s effectiveness in reducing HCC levels, though sex trends suggest potential differences in cortisol responses that warrant further investigation.

### 4.3. HCC Group Comparisons to Pre-COVID Normative Levels

The intervention group received four 15 min recess breaks daily from kindergarten through 3rd grade, followed by reducing their amount of recess daily to three 15 min recess breaks in the fall of this study after coming back from COVID to regain content missed from the previous semester. Although recess minutes daily were reduced for the intervention children by 15 min from previous years, at least 45 min of recess daily returning from the COVID break indicates children are less stressed than pre-COVID normative values and children who receive 30 min of recess daily. The intervention children’s significantly lower cortisol levels may be a byproduct of more time outdoors, going back to the pre-COVID school routine of recess breaks throughout the day, and providing children the opportunity to experience far more physical activity throughout each day than when they were isolated at home during COVID.

Research has shown repeatedly that unstructured, outdoor play, physical activity, and socialization are a means to lower stress levels in childhood [12,20,37,38]. This initial study showed that 45 min of recess can reduce chronic stress in children, but 30 min of recess may still play a role in decreasing stress levels compared to schools that only provide 15 min or less of recess daily. With increased seat time, increased testing standards, and post-COVID stress trends towards adverse mental and physical health, these results preliminarily show that recess built into a school day three to four times daily can be a positive health marker for reducing chronic stress in 4th grade children.

### 4.4. Limitations

One limitation of the study was the unequal number of children by age. Although there were no group differences by age, it would enhance the findings to know if different age groups do affect hair cortisol levels. Another limitation is that this was a quasi-experimental design assessing intervention and control groups with only one period of data collection in the fall. This study was focused on determining the initial results for chronic stress in children to determine the impact of more recess daily. Without a second period of data collection in the spring, it is harder to determine if something like standardized tests may cause chronic stress levels to rise higher than what the fall stress levels reflect. Another limitation was the lack of other demographic variables that could have been examined, such as socioeconomic status (SES) and race/ethnicity. SES, race, and ethnicity data were not available for this preliminary study and could impact hair cortisol results. Including these demographic variables in the future could enhance the findings even more. The last limitation is that we did not exclude children from having their hair assessed who had diseases that could influence the glucocorticoid levels (especially Addison’s disease and Chushing’s disease), including anxiety and attention-deficit/hyperactivity disorder), and pharmacological treatments (administered by any method, i.e., oral, inhaled, or topical) that could influence cortisol levels, including antipsychotics, ADHD medications, and corticosteroids. We did, however, minimize this risk by removing any child from the study who registered HCC levels two or more standard deviations away from the group mean.

### 4.5. Future Studies

Future research should explore how recess interventions, such as the LiiNK Project, impact children of other grades, races, and socioeconomic statuses, as well as longitudinally for body composition and hair cortisol. Future studies may explore the potential relationship between hair cortisol concentrations and daily somatic complaints, especially among the younger grade levels. Also, future research should compare chronic stress levels to bi-annual and longitudinal academic performance. There is also a vast need for research on learning barriers and interventions for children of all ages. Too often, it is assumed that conducting adult or parental report research provides a clear picture of what is happening to and within children. More HCC (chronic stress) research examining fall and spring data is necessary to reflect when stress is higher and who is impacted the most, i.e., boys or girls, different races, different grade levels. Finally, future research should examine stress differences between children with no anxiety/stress-related diagnoses and those with anxiety/stress-related diagnoses). Furthermore, other quantitative tools or qualitative methods should be added that assess these two groups to determine if there are strong relationships among increased outdoor play, physical activity, social and emotional health, and chronic stress, as other studies have shown [8,37,38].

## 5. Conclusions

The purpose of this study was to measure the effect of a recess intervention on children’s chronic stress levels. The intervention school children were closely matched with the control school children on most factors, including school type (public), demographics, family socioeconomic status, and teacher-to-student ratio, except for the inclusion of the LiiNK Project intervention throughout their school day. From the preliminary data collected in this study, fourth grade children receiving the LiiNK intervention’s three 15 min recesses daily were significantly less stressed than those who received two 15 min recess breaks daily.

This study is among the first to examine how unstructured outdoor recess breaks throughout the school day influence a child’s chronic stress levels. It is evident there are an abundant number of benefits to physical and psychological development and well-being from being physically active and socialized through recess. However, these results provide evidence that increasing recess by another 15 min during the school day contributes to lower chronic stress levels among elementary school children, as well as providing ample opportunities for children to be physically active and socialized daily. With obesity and mental health disorders on the rise, as well as United States students’ academic performance on the decline, these pilot results cannot be overlooked or dismissed.

## Data Availability

The dataset presented in this article is not readily available because the data are part of an ongoing study. Requests to access the datasets should be directed to Debbie Rhea.
